# Ethnicity and socioeconomic status do not influence glycaemic outcomes of a tubeless hybrid closed‐loop system (Omnipod® 5) in adults with type 1 diabetes

**DOI:** 10.1111/dom.16553

**Published:** 2025-07-09

**Authors:** Ananthi Anandhakrishnan, Siobhan Pender, Thomas Johnston, Rebecca Hyslop, Yuk‐Fun Liu, Dulmini Kariyawasam, Anna Brackenridge, Janaka Karalliedde, Sufyan Hussain

**Affiliations:** ^1^ Department of Diabetes and Endocrinology Guy's Hospital, Guys and St Thomas' NHS Trust London UK; ^2^ Department of Diabetes, School of Cardiovascular, Metabolic Medicine and Sciences King's College London London UK; ^3^ Institute of Diabetes, Endocrinology and Obesity King's Health Partners London UK

**Keywords:** ethnic diversity, hybrid closed loop system, publicly funded healthcare system, real‐world evidence, socioeconomic deprivation, type 1 diabetes

## Abstract

**Aims:**

We aimed to evaluate real‐world glycaemic outcomes of a tubeless hybrid closed‐loop (HCL) insulin delivery system in type 1 diabetes (T1D), exploring the influence of ethnicity and socioeconomic status from a publicly funded system with universal access.

**Materials and Methods:**

This was a retrospective observational study in adults with T1D initiating HCL (Omnipod® 5) at a large publicly funded multi‐site diabetes service. Baseline glycaemic metrics were compared with 12‐week post‐initiation data. Clinical data and social determinants of health, such as ethnicity and socioeconomic deprivation indices, were analysed for subgroup differences.

**Results:**

One hundred and sixty adults with T1D were included (26.9% non‐White; 48.8% in the two most deprived IMD quintiles). Mean time in range (%TIR; 3.9–10 mmol/L) improved from 52.7 ± 16% to 67.9 ± 12.7% (*p* < 0.001). Improvements were consistent across ethnic groups (mean % TIR +14.8% [95% CI: (12.6%, 17.3%)] and + 16.1% [95% CI: 12.4%, 19.7%] in White and non‐White individuals, respectively, *p* = 0.488) and socioeconomic strata (mean % TIR + 15.9% [95% CI: 13.3%, 18.6%] and + 14.2% [95% CI: 11.2%, 17.1%] in those from lower and higher socioeconomic groups, respectively, *p* = 0.290). Those with poorer baseline glycaemia experienced greater improvements.

**Conclusions:**

Early real‐world use of a tubeless HCL system demonstrated significant and equal glycaemic improvements in diverse ethnic and socioeconomic groups. Promoting universal access to HCL technologies in T1D is therefore essential to ensure existing disparities in glycaemic outcomes are minimised.

## 1 INTRODUCTION

Hybrid closed‐loop (HCL) insulin delivery systems represent a major advance in type 1 diabetes (T1D) management, automating insulin adjustments using real‐time glucose data.^1^ These systems consistently improve time in range (TIR) and reduce glycaemic variability with positive impacts on diabetes management burden in clinical trial^2^, ^3^, ^4^, ^5^, ^6^, ^7^, ^8^, ^9^, ^10^, ^11^ and real‐world^12^, ^13^, ^14^, ^15^, ^16^, ^17^, ^18^ settings. Hence, HCL systems are now considered the standard of care for T1D and have recently been endorsed by funding bodies in England with a widescale implementation programme.^19^

There are currently four commercially available HCL systems for use in the United Kingdom (UK). The Omnipod® 5 (OP5) is a tubeless HCL system and is also the latest commercial system to be introduced. Tubeless HCL systems offer advantages by eliminating infusion tubing, which may improve device acceptance and usability for individuals with T1D.^20^ Two pivotal multicentre non‐randomised clinical trials in the United States (US)^10^, ^11^ first reported the safety and efficacy of the OP5 system in adults and children with T1D. Following this, a large real‐world dataset in the US, where healthcare is primarily insurance‐based, reported efficacy of the system in 69 902 adults and children with T1D.^16^ In the UK, where healthcare is primarily publicly funded by the National Health Service (NHS), real‐world efficacy of the OP5 system has also recently been demonstrated in adults^21^, ^22^ and children.^23^ However, access in underserved groups remains an issue, even in publicly funded systems.^24^, ^25^ Owing to this, prior efforts have been unable to explore the translation of benefits from HCL in ethnic and socioeconomically deprived groups.

Factors including ethnicity, socioeconomic status, and differential access to technology persistently influence diabetes outcomes.^26^, ^27^, ^28^ Limited digital and health literacy have also been suggested as factors that could hinder optimal use of technology‐based treatments in certain groups.^29^, ^30^ Despite this, real‐world data in diverse populations is limited, with minority ethnic groups and deprived populations often underrepresented in clinical trials,^31^ and real‐world uptake for pumps remaining unequal as well.^24^, ^25^, ^32^, ^33^

Following a recent national implementation drive and local programme to promote access to HCL in underserved communities, there is a unique opportunity to explore the relationship between social determinants of health and HCL outcomes. We aimed to assess the influence of ethnicity and socioeconomic status on glycaemic outcomes following HCL initiation in a large, diverse cohort of adults with T1D attending an urban multi‐site diabetes service. Understanding the extent to which HCL systems improve glucose metrics across diverse groups can help identify barriers and inform targeted interventions to ensure equitable benefit.

## 2 METHODS

### 2.1 Study design

This retrospective cohort study included adults (≥18 years) with T1D initiating a tubeless HCL system (OP5) between July 2023 and June 2024 at a large multi‐site diabetes service, offering multiple specialist type 1 diabetes clinics at two different urban locations in London. Inclusion criteria were prior continuous glucose monitoring (CGM) use and availability of pre‐ and post‐initiation CGM data. Pregnant individuals or individuals who conceived at data collection time points, those without a coded ethnicity and those who discontinued HCL prior to 12 weeks of use were excluded from data analysis. Data collection took place between 17 April 2024 and 4 February 2025. This retrospective study was conducted in line with local institutional audit protocols using existing anonymised routine clinical data accessed directly by the clinical team.^34^

Glycaemic data were derived from device‐specific databases (e.g., Dexcom Clarity, Libreview, Glooko). Baseline CGM‐derived metrics were collected for a 14‐day period from the final sensor upload prior to HCL initiation. Post‐HCL metrics were taken 12 weeks after initiation, for a 14‐day period. Laboratory HbA1c, weight, and BMI data reflect comparisons between measurements obtained up to 12 months prior to and following HCL start.

Sensor glucose outcomes are reported as percentage time in range (TIR) [3.9–10.0 mmol/L (70–180 mg/dL)], percentage time above range (TAR) level 1 [10.0–13.9 mmol/L, (180–250 mg/dL)], percentage TAR level 2 [>13.9 mmol/L, (>250 mg/dL)], percentage time below range (TBR) level 1 [3.0–3.9 mmol/L, (54–70 mg/dL)] and percentage TBR level 2 [<3.0 mmol/L (<54 mg/dL)]. The glucose variability (coefficient of variation in percentages) and glucose management indicator (GMI) were also collected. Insulin delivery data collected includes the total number of insulin units delivered daily (based on device download or declared doses for those on MDI).

Descriptive data collected included age, gender, ethnicity, date of diagnosis with diabetes, attendance to structured education. Ethnicity is grouped as ‘White’ and ‘non‐White’ to include individuals coded on health records as: ‘White‐British’ and ‘White‐other’; and those coded as ‘Asian or Asian‐British’, ‘Black and Black‐British’, ‘Mixed’ or ‘Other’, respectively. Postcode‐based English Index of Multiple Deprivation (IMD) data was collected and is reported as a relative measure of socioeconomic status.^35^ Deciles are measured on a scale from one to ten, with lower values corresponding to greater levels of deprivation. Socioeconomic status is grouped as ‘lower’ and ‘higher’ to include individuals residing in IMD deciles: at or below the median IMD decile; and those residing above the median IMD decile, respectively.

The primary outcome measure was the change in sensor‐derived TIR. Secondary outcome measures included changes in: sensor‐derived TAR, TBR, GMI; laboratory‐derived HbA1c, total daily insulin dose and anthropometrics. The proportion meeting international consensus targets (TIR >70% and TBR <4%)^36^ and HbA1c/GMI targets of ≤6.5% as defined by NICE,^37^ ≤7.0% as defined by the American Diabetes Association (ADA),^38^ and ≤7.5%^39^ (HbA1c threshold for HCL funding in England^19^) were also assessed.

### 2.2 Statistical methods

Data is summarised using descriptive statistics and is reported as mean ± standard deviation (SD) and median [interquartile range (IQR)] unless otherwise stated. Distribution was determined by Kolmogorov–Smirnov tests of normality. Paired sample t‐tests or Wilcoxon signed‐rank tests were used to compare paired outcome measures. Independent sample t test or Mann–Whitney U tests were used to compare unpaired outcome measures. The Kruskal‐Wallis test was used to compare differences between three or more groups. For categorical variables, chi‐squared tests were used to determine differences in proportions between groups.

Spearman correlation coefficients were used to determine the relationships between categorical data and continuous data that did not follow a normal distribution, with the correlation coefficient R shown. Bivariate analysis with pairwise case exclusion was conducted to examine the individual relationships between baseline HbA1c, weight, sex, age, duration of diabetes, pump use, deprivation level, ethnicity and change in weight with change in TIR. A multivariable linear regression analysis with pairwise case exclusion was then performed using variables of interest to assess their combined effect on the primary endpoint. Standardized beta coefficient (B) is shown with 95% confidence intervals (95% CI).

Significance threshold for *p*‐values was <0.05. All *p*‐values were two‐sided. Statistical analyses were performed using SPSS version 29.0.2.0 (IBM Corp.).

## 3 RESULTS

One‐hundred and sixty participants met inclusion criteria (Supplementary Figure S1). 26.9% were non‐White and 48.8% resided within the two most deprived IMD quintiles. Non‐White participants were younger (*p* = 0.006) and tended to be from more deprived areas than White participants. Baseline mode of insulin delivery and prior diabetes‐structured education uptake did not differ significantly between ethnic and socioeconomic groups. Baseline characteristics of the total cohort are summarised in Table 1.

**TABLE 1:** Baseline characteristics of cohort.

Characteristic	Total cohort (*n* = 160)	Characteristics grouped by reported ethnicity			Characteristics grouped by socioeconomic status (SES)		
		White (*n* = 117)	Non‐White (*n* = 43)	*p*	Lower SES (IMD ≤ 5; *n* = 94)	Higher SES (IMD > 5; *n* = 66)	*p*
Age, years	38.6 ± 11.7, 36 [29.3, 46.6]	40.2 ± 11.7, 37 [32, 47.5]	34.5 ± 11, 33 [26, 43]	0.006	37.6 ± 11.5, 35.5 [29, 46]	40.1 ± 12, 37 [31, 46.25]	0.191
Diabetes duration, years	21.2 ± 10.8, 21 [14, 27.8]	22.6 ± 10.8, 22 [15, 29]	19.6 ± 10.6, 19 [11, 26]	0.102	22.6 ± 10.8, 22 [15, 29]	19.6 ± 10.6, 19 [11, 26]	0.354
Attendance to structured education							
Yes, *n* (%)	70 (41.6%)	52 (41.6%)	18 (41.9%)	*X* ^2^ **=** 0.09 *p* **=** 0.77	41 (43.6%)	29 (43.9%)	*X* ^2^ **=** 0.002 *p* **=** 0.968
No or unknown *n* (%)	90 (58.4%)	65 (58.4%)	25 (58.1%)		53 (56.4%)	37 (56.1%)	
Sex							
Female, *n* (%)	101 (60.6%)	73 (58.4%)	28 (66.7%)	*X* ^2^ **=** 0.1 *p* **=** 0.752	59 (62.8%)	42 (63.6%)	*X* ^2^ **=** 0.968 *p* = 0.637
Male, *n* (%)	59 (39.4%)	44 (41.6%)	15 (34.3%)		35 (37.2%)	24 (36.4%)	
Ethnicity							
White, British, *n* (%)	95 (59.4%)	95 (81.2%)	–		49 (52.1%)	46 (49.7%)	*X* ^2^ **=** 19.9 *p* **=** 0.001
White, other, *n* (%)	22 (13.8%)	22 (17.8%)	–		15 (16%)	7 (10.6%)	
Mixed, *n* (%)	3 (1.9%)	–	3 (7%)		2 (2.1%)	1 (1.5%)	
Asian or Asian British, *n* (%)	10 (6.3%)	–	10 (23.3%)		2 (2.1%)	8 (12.1%)	
Black or Black British, *n* (%)	18 (11.3%)	–	18 (41.9%)		17 (18.1%)	1 (1.5%)	
Other, *n* (%)	12 (7.5%)	–	12 (27.9%)		9 (9.6%)	3 (4.5%)	
Socioeconomic status							
Index of multiple deprivation decile; median [IQR]	5 [3,7]	5 [3,7]	3 [3,6]	*X* ^2^ **=** 4.2 *p* **=** 0.332	3 [3,4]	7 [6,9]	
IMD Quintile 1 (Most deprived)	17 (10.2%)	13 (11.1%)	4 (9.3%)		17 (18.1%)	–	
IMD Quintile 2	61 (38%)	39 (33.4%)	22 (51.2%)		61 (64.9%)	–	
IMD Quintile 3	34 (21.2%)	27 (23.1%)	7 (16.3%)		16 (17%)	18 (27.3%)	
IMD Quintile 4	20 (11 %)	15 (12.8%)	5 (11.6%)		–	20 (30.3%)	
IMD Quintile 5 (Least deprived)	28 (19.7%)	23 (19.7%)	5 (11.6%)		–	28 (42.4%)	
Baseline insulin delivery, *n* (%)							
MDI	54 (40.1%)	37 (31.6%)	17 (39.5%)	*X* ^2^ **=** 0.88 *p* **=** 0.348	36 (38.3%)	18 (27.3%)	*X* ^2^ **=** 2.108 *p* **=** 0.147
CSII (SAP)	106 (59.9%)	80 (63.4%)	26 (60.5%)		58 (61.7%)	48 (72.7%)	

*Note*: Data expressed as mean ± SD, median [IQR] unless otherwise specified.

Abbreviations: CSII, continuous subcutaneous insulin infusion; IMD, Index of multiple deprivation; MDI, multiple daily injection; *n*, number of individuals; SAP, sensor augmented pump; X^2^, chi‐squared statistic.

### 3.1 Improvement in glycaemic outcomes across the cohort with HCL

**FIGURE 1:**
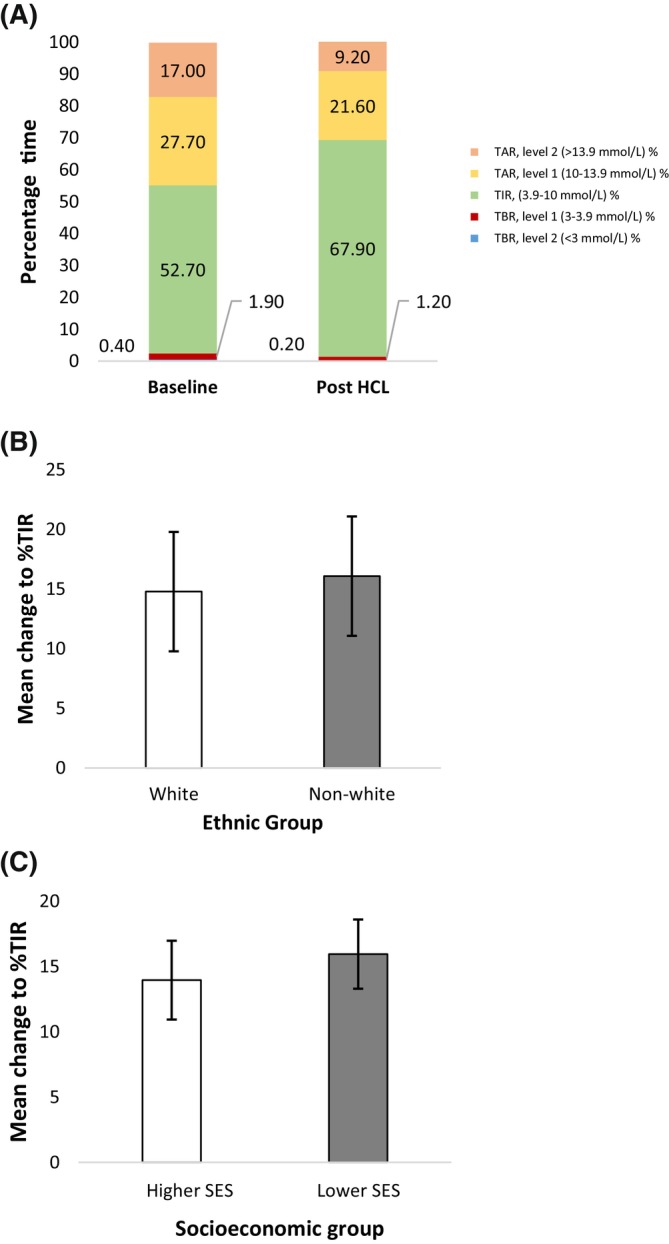
Glycaemic outcome measures post‐HCL: (A) percentage time in range (TIR) and time above range (TAR) (time >10 mmol/L) and time below range (TBR) (time <3 mmol/L) across total cohort; (B) Mean change to % time in range (TIR; 3–10 mmol/L) post‐HCL within ethnic groups, *p* < 0.001 for both groups; (C) mean change to % TIR within socioeconomic status (SES) groups, *p* < 0.001 for both groups. *HCL, hybrid closed loop; GMI, Glucose Management Indicator; TIR, Time in range; TAR, time above range; TBR, time below range*.

Across the cohort, significant improvements in sensor‐derived glucose metrics were observed following HCL (Table 2). These included a significant improvement in mean %TIR from 52.7 ± 16% to 67.9 ± 12.7% (*p* < 0.001) alongside reductions in time spent in level one and two hyper‐ and hypoglycaemia, summarised in Figure 1A. Mean GMI declined from 7.6 ± 0.8% to 7.2 ± 0.7% (*p* < 0.001). Similarly, laboratory HbA1c, available for 87% of the cohort (*n* = 139) significantly reduced from 7.7 ± 1.2% to 7.2 ± 0.8% (*p* < 0.001). Median time in auto mode was 99% [96–100].

**TABLE 2:** Baseline and post‐HCL outcomes for cgm and laboratory glucose measures, weight, BMI and insulin use: total cohort baseline and post‐HCL analysis and comparisons of average change by ethnicity and socioeconomic status (SES).

Characteristic, unit of measure (*n*)	Total cohort (*n* = 160)				Outcome measures compared between ethnic and socioeconomic status (SES) groups					
					Ethnic groups			Socioeconomic groups		
					Average change (95% CI)			Average change (95% CI)		
	Baseline	Post‐HCL	Average change (95% CI)	*p*‐value	White (*n* = 117)	Non‐White (*n* = 43)	*p*‐value	Lower SES (IMD ≤5; *n* = 94)	Higher SES (IMD >5; *n* = 66)	*p*‐value
CGM metrics										
GMI, %	7.6 ± 0.8, 7.6 [7.1, 8.1]	7.2 ± 0.7, 7.2 [6.9, 7.4]	−0.4 (−0.5, −0.4)	<0.001	−0.5 (−0.6, −0.4)	−0.4 (−0.6, −0.3)	0.907	−0.5 (−0.6, −0.3)	−0.4 (−0.5, −0.3)	0.519
TAR, level 2 (>13.9 mmol/L, >250 mg/dL) %	17 ± 13.5, 13.5 [7, 24.8]	9.2 ± 9.4, 7 [3.3, 12]	−7.9 (−9.5, −6.2)	<0.001	−8 (−9.9, −6)	−7.6 (−10.6, −4.5)	0.620	−8.4 (−10.6, −6.1)	−7.1 (−9.3, −4.9)	0.784
TAR, level 1 (10–13.9 mmol/L,180–250 mg/dL) %	27.7 ± 7.9, 27 [23.3, 33]	21.6 ± 6.6, 22 [17, 25]	−6 (−7.3, −4.8)	<0.001	−5.6 (−7.2, −4)	−7.2 (−9.3, −5)	0.259	−6.2 (−7.9, −4.5)	−5.8 (−7.7, −4)	0.344
TIR, (3.9–10 mmol/L, 70–180 mg/dL) %	52.7 ± 16, 53 [42, 65]	67.9 ± 12.7, 69.5 [61.3, 76]	15.1 (13.3, 17.2)	<0.001	14.9 (12.6, 17.3)	16.1 (12.4, 19.7)	0.488	15.9 (13.3, 18.6)	14.2 (11.2, 17.1)	0.290
TBR, level 1 (3–3.9 mmol/L, 70–54 mg/dL) %	1.9 ± 2.2, 1 [0, 3]	1.2 ± 1.1, 1 [0,2]	−0.8 (−1.1, −0.5)	<0.001	−0.8 (−1.1, −0.4)	−0.8 (−1.5, −0.16)	0.959	−0.8 (−1.1, −0.4)	−0.8 (−1.3–0.3)	0.630
TBR, level 2 (<3 mmol/L, 54 mg/dL) %	0.4 ± 0.9, 0 [0,0]	0.2 ± 0.5, 0 [0,0]	−0.2 (−0.3, −0.02)	0.034	−0.1 (−0.3, 0.04)	−0.3 (−0.6, 0.1)	0.292	−0.1 (−0.3, 0.1)	−0.2 (−0.5, 0.01)	0.514
Average glucose, mmol/L	10.2 ± 2.7, 9.9 [8.8, 11.1]	9 ± 1.3, 8.9 [8.4,9.6]	−1.2 (−1.6, −0.8)	<0.001	−1.2 (−1.7, −0.7)	−1 (−1.4, −0.6)	0.755	−0.6 (−1.4, −0.8)	−1.4 (−2.2, −0.5)	0.482
Coefficient of variation, %	36.3 ± 6.2, 35.8 [33.1,40.3]	33.8 ± 4.6, 34 [30.4, 36.7]	−2.5 (−3.5, −1.5)	<0.001	−3 (−4.2, −1.6)	−1.2 (−3, 0.7)	0.360	−2.1 (−3.4, −0.8)	−3 (−4.8, −1.4)	0.054
Laboratory HbA1c, anthropometrics and insulin dosing										
HbA1c % *(n, %)*	7.7 ± 1.2, 7.6 [6.8, 8.4] ((*n = 139, 87%*)	7.2 ± 0.8, 7.1 [6.6, 7.5]	−0.53 (−7, −0.4)	<0.001	−0.5 (−0.6, −0.3)	−0.7 (−0.9, −0.5)	0.067	−0.6 (−0.8, −0.4)	−0.5 (−0.7, −0.2)	0.289
Weight, kg *(n, %)*	74.9 ± 13.4, 72.5 [66, 85.6] (*n = 140, 87%*)	76 ± 14.7, 73 [65.6, 85.5]	1.1 (0.32, 1.82)	0.008	0.9 (0.01, 1.8)	1.6 (0.1, 3.0)	0.269	0.8 (−0.2, 1.9)	1.25 (0.2, 2.3)	0.897
BMI, kg/m^2^ *(n, %)*	26.1 ± 3.9, 25.5 [23.8, 28] (*n = 140, 87%*)	26.2 ± 4.3, 25.7 [23.6, 28.5]	0.17 (−0.14, 0.5)	0.201	0.1 (−0.3, 0.5)	0.4 (−0.2,1)	0.268	0.1 (−0.2, 0.5)	0.2 (−0.3, 0.7)	0.521
Total daily insulin dose, units	39.06 ± 13, 36.8 [30.1, 56.7]	41.6 ± 19.9, 37.4 [28.6, 50.6]	2.5 (−0.04, 5.2)	0.133	0.5 (−5, 7.7)	1.8 (−6.5, 11.6)	0.424	0.5 (−1, 7.3)	1.8 (−0.8, 4.3)	0.954

*Note*: Data expressed as mean ± SD, median [IQR] unless otherwise specified. *n* = 160 unless otherwise specified.

Abbreviations: CGM, Continuous glucose monitor; CSII, Continuous subcutaneous insulin infusion; GMI, Glucose Management Indicator; HbA1c, glycated haemoglobin; HCL, hybrid closed loop; MDI, Multiple daily dose insulin; *n*, number of individuals; SAP, sensor augmented pump therapy; TAR, time above range; TBR, time below range; TIR, Time in range.

The proportion meeting international TIR targets increased from 5% (*n* = 8/160) to 38.8% (*n* = 62/160) post HCL (*p* < 0.001). Following HCL initiation, the proportion attaining GMI ≤6.5%^37^ non‐significantly improved (*p* = 0.214). These individuals made up less than 10% of the total cohort (*n* = 12/160). 39% of the total cohort attained a GMI ≤7.0%^38^ post HCL (*n* = 63/160), a relative 80% increase from baseline (*n* = 35/160) (*p* < 0.001). Of the total cohort, 77.5% attained a GMI ≤7.5%^39^ 40 (*n* = 124/160) representing a relative increase of 73% from baseline (*n*=72/160; *p* < 0.001) (Figure S2A).

**FIGURE 2:**
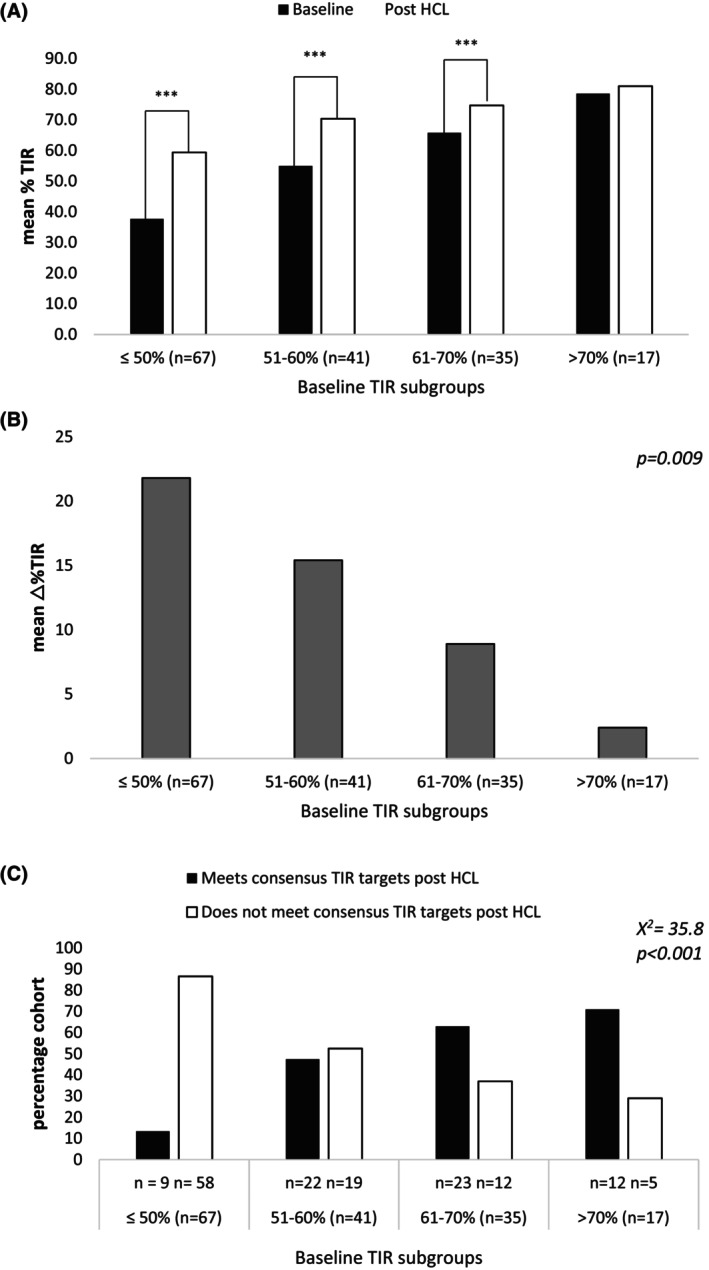
% Time in range (TIR) improvements and consensus TIR attainments at baseline and post‐HCL in total cohort grouped according to baseline glycaemic control. 2A; Significant improvement in mean TIR post‐hybrid closed‐loop (HCL) was seen in all subgroups with the exception of those with baseline TIR >70%. 2B; Lower baseline TIR was associated with greater improvements to TIR. 2C; the likelihood of attaining consensus TIR targets post‐HCL was related to baseline glycaemic control. TIR; time in range, △; change.

Individuals were grouped according to baseline TIR; those with baseline TIR ≤50% (*n* = 67), baseline TIR 51–60% (*n* = 41) and baseline TIR 61%–70% (*n* = 35) and baseline TIR >70% (*n* = 17). Significant improvements to mean TIR were seen within all subgroups except for those with baseline TIR >70% (*p* = 0.214) (Figure 2A). Whilst greater improvements to TIR were seen in those with lower baseline TIR (*p* = 0.009) (Figure 2B), those with higher baseline TIR were more likely to attain consensus TIR targets post‐HCL (*p* < 0.001, Figure 2C).

There was a modest increase in weight across the cohort (mean weight: +1.2 ± 1 kg, *p* = 0.008) without a significant increase in BMI and a non‐significant trend towards higher insulin doses (baseline: 0.5 ± 0.2 units/kg; follow‐up: 0.5 ± 0.3 units/kg, *p* = 0.133) (Table 2).

### 3.2 Ethnicity and socioeconomic status do not influence glycaemic improvements from HCL

**FIGURE 3:**
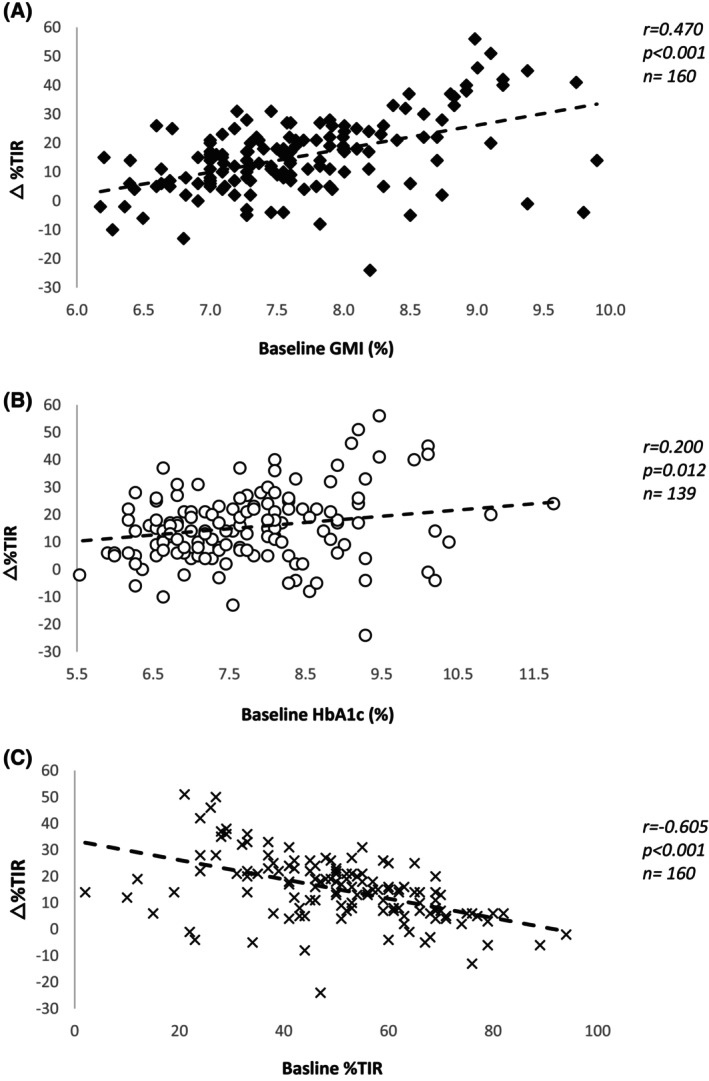
Baseline glycaemic control influences improvement to % time in range (TIR) following the use of hybrid closed‐loop (HCL). Baseline glucose management indicator (GMI) (3A) and baseline glycated haemoglobin HbA1c (3B) were positively correlated with improvements to TIR post‐HCL, with a strong negative correlation seen between baseline TIR (3C) and improvements to TIR post HCL. △, change.

Bivariate analyses were performed and demonstrated a significant association between baseline GMI (R = 0.470, *p* < 0.001), TIR (R = −0.605, *p* < 0.001) and HbA1c (R = 0.209, *p* = 0.008) with the primary endpoint. We observed that those with poorer baseline glycaemic control experienced greater glycaemic improvements following HCL (Figure 3). Bivariate analyses did not demonstrate any significant association between ethnicity or socioeconomic status with the primary endpoint (Supplementary Table S1). We observed an association between coded ethnicity with baseline TIR (R = 0.173, *p* = 0.029) and HbA1c (R = −0.192, *p* = 0.015). No association was observed between coded socioeconomic status with baseline GMI, TIR or HbA1c (each of the three variables identified as significantly associated with the primary outcome measure). When socioeconomic status was grouped, as ‘lower’ or ‘higher’ (see Methods) an association was observed between this, and baseline TIR (R = 0.157, *p* = 0.047) (Supplementary Table S2).

Baseline TIR (B = −1.114, 95% CI [−1.102, −0.652], *p* < 0.001), baseline HbA1c (B = −0.272, 95% CI [−0.473, −0.111], *p* = 0.002) and baseline GMI (B = −0.325, 95% CI [=0.946, −0.034], *p* = 0.035) remained independently associated with the primary end‐point in multivariable linear regression analysis. There were no statistically significant associations observed between ethnicity or socioeconomic status with the primary endpoint when entered into the multivariable model (Supplementary Table S3).

### 3.3 Improvement in glycaemic outcomes are similar between ethnic groups

Significant improvements in sensor‐derived glucose metrics and laboratory HbA1c were observed in both White and non‐White individuals following HCL. Glycaemic improvements were comparable across ethnicities and included a significant improvement in mean %TIR of +14.9% (95% CI: 12.6%, 17.3%) in White individuals, and + 16.1% (95% CI: 12.4%, 19.7%) in non‐White individuals, respectively, *p* = 0.488 (Figure 1B, Table 2).

At baseline, glycaemic variability was similar between White and non‐White individuals (*p* = 0.325). However, following HCL non‐White individuals had greater glycaemic variability (*p* < 0.001) and spent more time in level two hyperglycaemia (*p* = 0.016) compared with their White counterparts. This was observed alongside a significantly reduced glycaemic variability in White individuals alone; *p* < 0.001. Supplementary Table S4 reports baseline and post‐HCL outcome measures compared between ethnic groups. Other than this change, similar patterns of glycaemic improvement were observed within White and non‐White individuals when outcome measures within ethnic groups were compared as baseline versus post‐HCL. These are summarised in Supplemental Figure S2B and detailed in Supplemental Table S5.

Change in HbA1c post‐HCL showed a trend towards a greater reduction in non‐White versus White individuals; however, this did not reach statistical significance. The mean reduction in HbA1c was −0.5% (95% CI: −0.6%, −0.3%) in White individuals compared with −0.7% (95% CI: −0.9%, −0.5%) in non‐White individuals, *p* = 0.067 (Table 2).

### 3.4 Improvement in glycaemic outcomes are similar between socioeconomic strata

Significant improvements in sensor‐derived glucose metrics and laboratory HbA1c were observed in both those from lower and higher socioeconomic backgrounds following HCL. Glycaemic improvements were comparable across socioeconomic groups and included a significant improvement in mean % TIR + 15.9% (95% CI: 13.3%, 18.6%) in those from lower socioeconomic groups and + 14.2% (95% CI: 11.2%, 17.1%) in those from higher socioeconomic groups, respectively, *p* = 0.290; (Figure 1C, Table 2).

At baseline, socioeconomic status was found to influence TIR, with those from lower socioeconomic backgrounds spending less TIR than their counterparts from higher socioeconomic backgrounds (*p* = 0.048). This difference was no longer observed post‐HCL (*p* = 0.082). Glycaemic variability was similar between groups at baseline (*p* = 0.787). Following HCL, despite significant reductions in glycaemic variability in both groups, those from lower socioeconomic backgrounds had greater glycaemic variability (*p* = 0.012) compared with those from higher socioeconomic backgrounds. This was observed alongside a greater time spent in level two hyperglycaemia (*p* = 0.014), higher average glucose (*p* = 0.039) and higher GMI (*p* = 0.042) in the same group post‐HCL. Supplemental Table S6 reports baseline and post‐HCL outcome measures compared between socioeconomic groups. Despite these observations, when outcome measures within socioeconomic groups were compared as baseline versus post‐HCL, similar patterns of glycaemic improvement were observed within individuals from lower and higher socioeconomic backgrounds, and is summarised in Supplemental Figure S2C and detailed in Supplemental Table S7.

Change in HbA1c post‐HCL showed a trend towards a greater reduction in those from lower socioeconomic backgrounds; however, this did not reach statistical significance (mean reduction in HbA1c −0.6% [95% CI: −0.8%, −0.4%]) in those from lower socioeconomic groups compared with −0.5% (95% CI: −0.7%, −0.2%) in those from higher socioeconomic backgrounds (*p* = 0.289) (Table 2).

## 4 DISCUSSION

This study demonstrates significant glycaemic improvements with an HCL system in a diverse publicly funded healthcare setting with universal access. Significant improvements in TIR, reductions in hyper‐ and hypoglycaemia, and decreases in HbA1c align with findings from controlled trials and large datasets primarily conducted in more homogenous populations.^40^

### 4.1 Ethnicity and socioeconomic status do not influence glycaemic outcomes following HCL

Previous research highlights that systemic disparities in diabetes outcomes are often tied to ethnic^41^, ^42^ and socioeconomic factors.^27^ In recent real‐world data, African Caribbean ethnicity was observed to be a significant independent risk factor for sight‐threatening diabetic retinopathy and progression of diabetic kidney disease.^41^, ^42^ Access to advanced diabetes technologies has historically followed similar patterns, with underrepresentation of minority and deprived populations.^32^, ^33^ Diabetes research has also been reported to be less representative of those from minority backgrounds,^31^ likely contributed to by reduced representation from these groups. It is of note, therefore, that 48.8% of our cohort belonged to the two most deprived quintiles and, compared with national census data on the general population representation in England and Wales, our cohort included a proportionate representation from individuals from non‐White backgrounds.^43^

Our data highlights the consistency of glycaemic improvements across ethnic groups and socioeconomic strata. Our findings suggest that when access is provided under a universal healthcare framework, these groups can achieve glycaemic improvements comparable with their White and less deprived counterparts. This supports the notion that differences in health outcomes are, at least in part, driven by unequal access rather than intrinsic patient factors.

Our cohort also highlighted greater hyperglycaemia and higher glycaemic variability post‐HCL in non‐White individuals compared with White individuals and those with lower socioeconomic status compared with those with higher socioeconomic status. Factors contributing to this observation require further study.

### 4.2 The unmet needs of hybrid closed loop systems

Our real‐world study also highlights that less than half of our cohort met international consensus TIR or recommended HbA1c/GMI targets post‐HCL. It also highlights that a very small proportion of individuals attained HbA1c/GMI targets of <6.5%,^37^ This underscores ongoing challenges when applying technologies in diverse real‐world settings, and additional efforts are needed towards achieving glycaemic targets and restoring normoglycaemia. Further studies are required to understand the reasons for differences in the efficacy of HCL systems in diverse real‐world populations with an emphasis on HCP and user interactions, as well as optimal device settings. It is possible that, in addition to further device developments, education and support tailored to the needs of individuals may be needed. Additionally, addressing usability concerns, previously identified barriers to technology uptake^44^ and algorithm improvements may further enhance real‐world benefits.

### 4.3 Strengths and limitations

This study's strengths include a relatively diverse cohort, with substantial inclusion of individuals from ethnic minority groups and socially deprived backgrounds from a publicly funded healthcare setting that eliminates financial barriers to access. Nevertheless, we highlight several limitations. We examined outcomes at 12 weeks. Whilst longer follow‐up may be desirable to support the durability of these improvements, recent data highlight that benefits from HCL noted at 12 weeks persist for up to 1 year.^22^ Furthermore, follow‐up data from individuals taking part in the OP5 pivotal trials^10^, ^11^ demonstrate that three‐month glycaemic outcomes are maintained for up to 2 years of at‐home use.^45^, ^46^

As this was a retrospective study based on available real‐world data, a formal prospective power calculation was not performed. However, we ensured that the sample size was adequate to address our primary objectives by including all eligible participants meeting inclusion criteria over the study period. Broader grouping of participants as White or non‐White and those from higher or lower socioeconomic status (see Methods) is unlikely to affect inferences; however, this approach may mask differences or differential contributions from ethnic and socioeconomic subgroups.

We relied on postcode‐based IMD as a proxy for socioeconomic status.^35^ Whilst validated, it remains a surrogate measure. The retrospective nature and lack of a control group limit our ability to infer causality. Clinical trials that explore the interactions between socioeconomic and ethnic demographics on glycaemic outcomes with diabetes technologies are needed to confirm our observations. Given appointment visit frequency in real‐world public funded model of healthcare, laboratory HbA1c and anthropometrics were up to 12 months before and post‐HCL initiation, respectively, and were not available for the total cohort.

Bivariate associations between ethnicity and baseline TIR and HbA1c, alongside observed associations between socioeconomic status and baseline TIR, indicate pre‐existing disparities in diabetes management across demographic groups. However, neither ethnicity nor socioeconomic status was associated with the primary endpoint, change in sensor‐derived TIR. Bivariate testing and multivariate analyses reconfirmed that ethnicity and socioeconomic status in our cohort do not influence glycaemic outcomes with HCL systems. This supports the efficacy of HCL and the importance of access across diverse populations. These analyses reinforce the concept that ethnicity and socioeconomic status in our cohort do not influence glycaemic outcomes with HCL systems. This supports the efficacy of HCL and the importance of access across diverse populations. Similar to other real‐world observations^40^, ^47^ and clinical trial data,^40^ the outcomes from the present study do not include CGM metrics such as time in tight range (3.9–7.8 mmol/L), which has recently been identified as a core endpoint for CGM‐derived glucose data for AID systems in clinical trials.^48^ We hope the results of this work will inform the design of future studies and clinical trials in this important area.

Despite these limitations, our data offer encouraging evidence that HCL technology can deliver substantial glycaemic improvements across diverse populations. Ensuring equitable access and providing targeted education and support are critical next steps. This data also reaffirms that those from groups at higher risk of complications should be prioritised to receive access to HCL systems. By integrating HCL systems into standard T1D care and proactively addressing barriers related to health/digital literacy, we have an opportunity to reduce disparities and improve outcomes for all, regardless of ethnicity or socioeconomic status. Future work needs to focus on the impact of HCL systems on patient satisfaction, quality of life and device acceptance with qualitative and patient‐reported outcomes studies.

## 5 CONCLUSIONS

In a UK real‐world setting, a tubeless HCL insulin delivery system significantly improved glycaemic measures in adults with T1D, regardless of ethnicity and socioeconomic deprivation. While universal access appears to mitigate some inequalities, unmet targets for many people with T1D highlight the need for further device and algorithmic improvements, as well as strategies that enhance engagement and support. By doing so, the full potential of HCL systems can be realised, moving closer to equitable diabetes care outcomes for all.

## AUTHOR CONTRIBUTIONS

A.A. and S.H. designed the study and conducted the clinical data extraction. A.A., S.H., and J.K. performed the analysis and co‐authored the first draft of the manuscript. All authors critically reviewed and approved the final version of the manuscript. S.H. is the guarantor of the work and, as such, had full access to all the data in the study and takes responsibility for the integrity of the data and the accuracy of the data analysis.

## FUNDING INFORMATION

S.H. is a recipient of the Medical Research Council Clinical Academic Partnership award (MR/W030004/1). A.A. is supported by an unrestricted educational grant from Abbott UK, awarded to SH. The research work was supported by an investigator‐initiated research grant from Insulet. The funders had no role in the design, conduct, analysis or reporting of the study findings.

## CONFLICT OF INTEREST STATEMENT

A.A. has no conflicts of interest to declare. S.H. has served on the advisory board for Tandem, Dexcom, Medtronic; undertaken non‐promotional educational and/or consultancy work for Abbott UK, Insulet, Dexcom, and Roche; received an unrestricted educational research grant from Abbott UK and an investigator‐initiated research grant from Insulet. All other authors report no conflict of interest.

## PEER REVIEW

The peer review history for this article is available at https://www.webofscience.com/api/gateway/wos/peer‐review/10.1111/dom.16553.

## Supporting information


**Data S1.** Supporting Information.

## Data Availability

The data that support the findings of this study are available from the corresponding author upon reasonable request.
