# Supplementary material for: Ethnicity and socioeconomic status do not influence glycaemic outcomes of a tubeless hybrid closed‐loop system (Omnipod® 5) in adults with type 1 diabetes
Source: Diabetes Obes Metab. 2025 Jul 9;27(9):5052–63. doi: 10.1111/dom.16553 (PMC12326946; doi:10.1111/dom.16553)
Supplement: Supplementary file 1 — Data S1. Supporting Information. [file DOM-27-5052-s001.pdf]

__Supplementary figures and tables__

Supplementary figure 1: Total cohort. Two-hundred and eight people with type 1 diabetes (pwT1D) commenced on the Omnipod® 5 (OP5) hybrid closed-loop (HCL) system from July 2023 and June 2024. Forty-eight individuals were excluded, leaving a total cohort of 160.

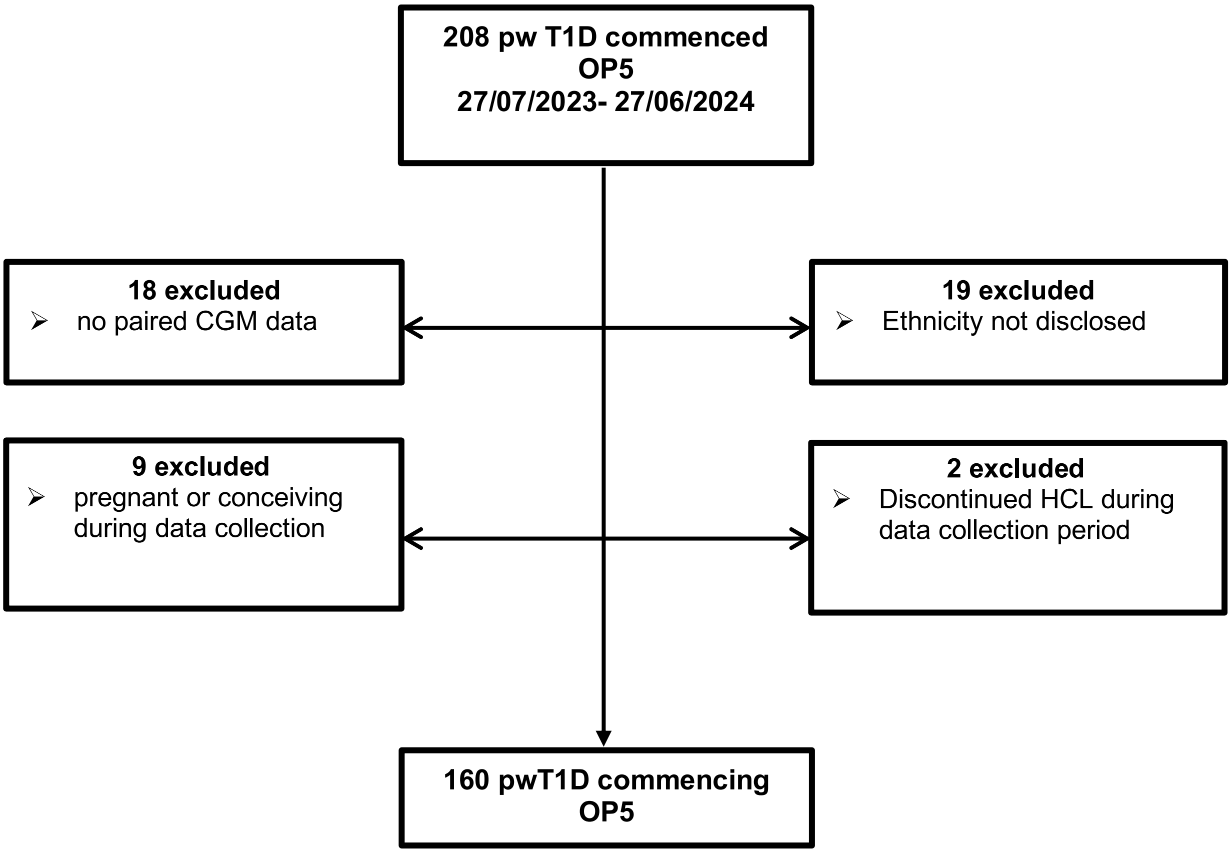

Supplementary figure 2: CGM derived glycaemic outcome measures: A, percentage cohort attaining GMI targets and consensus TIR targets in the total cohort represented as baseline and HCL; **B,** percentage TIR and TAR and TBR within ethnic groups post HCL; **C,** percentage TIR and TAR and TBR within SES groups post HCL. *CGM, Continuous glucose monitor; HCL, hybrid closed loop; GMI, Glucose Management Indicator; TIR, Time in range; TAR, time above range; TBR, time below range.*

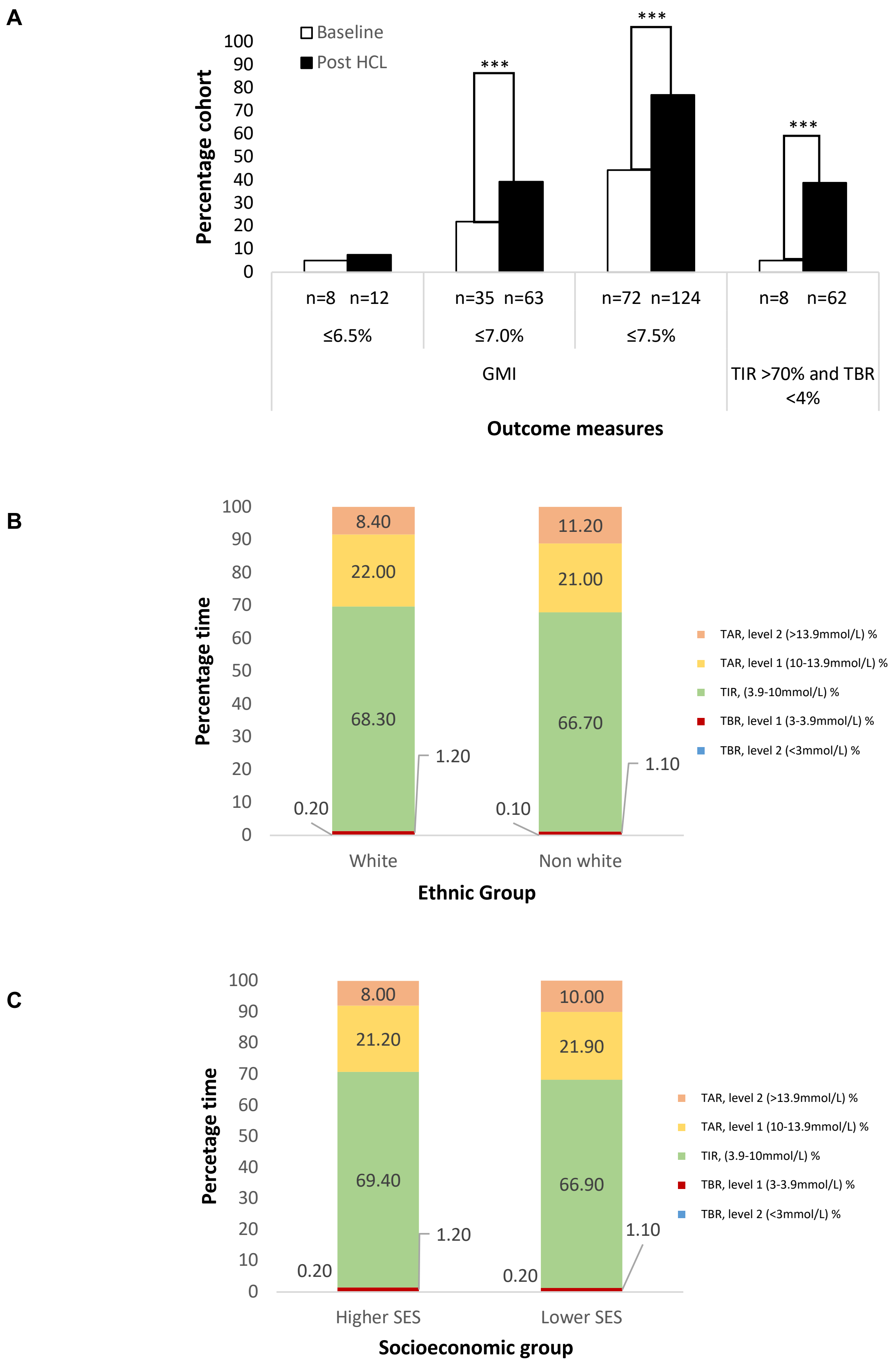

Supplementary table 1: Bivariate associations between independent variables of interest with primary endpoint of change in TIR.

Independent variables		Change in TIR	Independent variables		Change in TIR
**Gender**	Correlation Coefficient	.132	**Baseline GMI**	Correlation Coefficient	.470**
	Sig. (2-tailed)	.096		Sig. (2-tailed)	<.001
	N	160		N	160
**Age**	Correlation Coefficient	-.104	**Baseline CV**	Correlation Coefficient	-.013
	Sig. (2-tailed)	.189		Sig. (2-tailed)	.871
	N	160		N	160
**Duration of diabetes**	Correlation Coefficient	-.034	**Baseline TIR**	Correlation Coefficient	-.605**
	Sig. (2-tailed)	.667		Sig. (2-tailed)	<.001
	N	160		N	160
**IMD (deciles 1-10)**	Correlation Coefficient	.000	**Baseline Weight**	Correlation Coefficient	-0.001
	Sig. (2-tailed)	.996		Sig. (2-tailed)	.992
	N	160		N	157
**IMD (grouped as higher vs lower SES)**	Correlation Coefficient	-.084	**Baseline BMI**	Correlation Coefficient	.093
	Sig. (2-tailed)	.292		Sig. (2-tailed)	.245
	N	160		N	157
**Attendance to structured education**	Correlation Coefficient	0.026	**Baseline HbA1c**	Correlation Coefficient	.209**
	Sig. (2-tailed)	0.742		Sig. (2-tailed)	0.08
	N	160		N	159
**Ethnicity (coded)**	Correlation Coefficient	-.055	**Change in weight**	Correlation Coefficient	0.078
	Sig. (2-tailed)	.490		Sig. (2-tailed)	0.364
	N	160		N	140
**Ethnicity (grouped as white vs non-white)**	Correlation Coefficient	-.088	**Dependent variable: change in TIR**		
	Sig. (2-tailed)	.267			
	N	160			

n, number of individuals; GMI, Glucose Management Indicator; CV, Co-efficient of variation; TIR, Time in range; TAR, time above range; TBR, time below range; HbA1c, glycated haemoglobin; CGM, Continuous glucose monitor; MDI, Multiple daily dose insulin; CSII, Continuous subcutaneous insulin infusion; SAP, sensor augmented pump therapy. n= 160 unless otherwise specified

Supplementary table 2: Bivariate associations between three variables independently associate with primary outcome measure (baseline GMI, baseline TIR, baseline HbA1c) with ethnicity (A), IMD decile (B), grouped ethnicity as higher or lower socioeconomic status (SES) (C) and grouped ethnicity as white and non-white (D).

**Table 2A**					
			**Baseline GMI**	**Baseline TIR**	**Baseline HbA1c**
	**Coded ethnicity**	**Correlation Coefficient**	-.136	.173*	-.192*
		**Sig. (2-tailed)**	.087	.029	.015
		**N**	160	160	159
**Table 2B**					
			**Baseline GMI**	**Baseline TIR**	**Baseline HbA1c**
	**Coded IMD decile**	**Correlation Coefficient**	-.077	.080	-.055
		**Sig. (2-tailed)**	.335	.316	.494
		**N**	160	160	159
**Table 2C**					
			**Baseline GMI**	**Baseline TIR**	**Baseline HbA1c**
	**White vs Non-white**	**Correlation Coefficient**	-.064	.086	-.070
		**Sig. (2-tailed)**	.418	.279	.379
		**N**	160	160	159
**Table 2D**					
			**Baseline GMI**	**Baseline TIR**	**Baseline HbA1c**
	**Higher or lower SES**	**Correlation Coefficient**	-.128	.157*	-.132
		**Sig. (2-tailed)**	.106	.047	.096
		N	160	160	159

GMI, Glucose Management Indicator; TIR, Time in range; HbA1c, glycated haemoglobin; IMD, index of multiple deprivation; SES, socioeconomic status.

Supplementary table 3: Multivariate analyses results for primary endpoint of change in TIR (dependent variable) and variables associated with the primary endpoint from bivariate testing (baseline GMI, baseline TIR, baseline HbA1c), ethnicity and IMD as coded (3A) and grouped (3B).

**Table 3A**					
		**Beta**	**p value**	**95.0% Confidence Interval for B**	
				**Lower Bound**	**Upper Bound**
**Dependent variable: change in TIR**	**Coded ethnicity**	.016	.782	-0.898	1.191
	**Coded IMD**	.040	.494	-.385	.793
	**Baseline GMI**	-.325	.035	-.946	-.034
	**Baseline TIR**	-1.114	<.001	-1.102	-.652
	**Baseline HbA1c**	-.272	.002	-.473	-.111
**Table 3B**					
		**Beta**	**p value**	**95.0% Confidence Interval for B**	
				**Lower Bound**	**Upper Bound**
**Dependent variable: change in TIR**	**White vs Non-white**	.023	.706	-2.691	3.965
	**Higher or lower SES**	0.001	.984	-2.984	3.044
	**Baseline GMI**	-.327	.034	-.949	-.037
	**Baseline TIR**	-1.115	<.001	-1.104	-.652
	**Baseline HbA1c**	-.275	.001	-.463	-.114

GMI, Glucose Management Indicator; TIR, Time in range; HbA1c, glycated haemoglobin; IMD, index of multiple deprivation; SES, socioeconomic status.

Supplementary table 4: Baseline and post-HCL outcome measures compared between ethnic groups.

**Characteristic, unit of measure *(n)***	**Baseline**			**Post-HCL**		
	White (n=117)	Non-white (n=43)	p-value	White (n=117)	Non-white (n=43)	p-value
**CGM metrics**						
**GMI, %**	7.6 ± 0.8, 7.6 [7.1, 8.1]	7.7 ± 0.7, 7.7 [7.1, 8.1]	0.416	7.2 ± 0.6, 7.1 [6.9, 7.4]	7.3 ± 0.6, 7.2 [6.9, 7.5]	0.335
**TAR, level 2 (>13.9 mmol/L, >250mg/dl,) %**	16.4 ± 14, 11 [6.5, 23]	18.8 ± 12, 17 [8, 25]	0.132	8.4 ± 9.2, 6 [3, 12]	11.2 ± 9.8, 8 [5,14]	0.016
**TAR, level 1 (10-13.9 mmol/L,180-250mg/dl) %**	27.5 ± 8.6, 28 [23, 33]	28.2 ± 5.7, 27 [25, 31]	0.914	22 ± 7, 22 [17, 26.5]	21 ± 5.2, 21 [18, 25]	0.493
**TIR, (3.9-10 mmol/L, 70-180mg/dl) %**	53.4 ± 17, 53 [42, 66]	50.7 ± 13.1, 51 [43, 61]	0.278	68.3 ± 12.9, 70 [62, 76.5]	66.7 ± 12.9, 68 [60, 75]	0.350
**TBR, level 1 (3-3.9 mmol/L, 70-54mg/dl) %**	1.9 ± 2.2, 1[0,3]	2 ± 2.3, 1 [0,3]	0.962	1.2 ± 1.1, 1[0,2]	1.1 ± 1, 1 [0,2]	0.872
**TBR, level 2 (<3 mmol/L, 54mg/dl) %**	0.4 ± 0.9, 0 [0,0]	0.4 ± 0.9, 0 [0,1]	0.308	0.2 ± 0.6, 0 [0,0]	0.1 ± 0.4, 0 [0,0]	0.737
**Average glucose, mmol/L**	10.2 ± 1.5. 9.8 [8.8,11]	10.2 ± 1.5, 10.1 [8.7, 11.1]	0.382	9 ± 1.3, 8.9 [8.4, 9.6]	9.2 ± 1.4, 9 [8.4, 9.8]	0.380
**Coefficient of variation, %**	36±6.2, 35.5 [33.5, 39.6]	37.1 ± 6.2, 37.1 [32.7, 42.4]	0.325	33 ± 4.4, 32.7 [29.9, 35.9]	35.9 ± 4.5, 35.8 [33.7, 39.2]	<0.001
**Laboratory HbA1c, anthropometrics and insulin dosing**						
**HbA1c % *(n, %)***	7.7 ± 1.2, 7.4, [6.6, 8.3] *(n=102, 87%)*	7.9 ± 0.9, 7.9 [6.9, 8.6] *(n=37, 86%)*	0.134	7.2 ± 0.9, 7.1, [6.6, 7.6]	7.2 ± 0.6, 7.2 [6.9, 7.6]	0.606
**Weight, kg *(n, %)***	74.7 ± 13.2, 73.7 [65, 86.8] *(n=103, 87%)*	75.5 ± 13.9, 70.8 [67, 84.5] *(n=37, 86%)*	1.000	75.6 ± 14.3, 73.5 [64.5, 86.4]	77.1 ± 15.7, 74.4 [67.5, 85.9]	0.653
**BMI, kg/m^2 *(n, %)***	25.8 ± 4, 25.5 [23.4, 27.8] *(n=103, 87.2%)*	26.8 ± 3.8, 26.7 [24.3, 28.4] *(n=37, 86%)*	0.121	25.9 ± 4.2, 25.4 [23.1, 28.5]	27.2 ± 4.7, 26.8 [24.1, 29.2]	0.131
**Total daily insulin dose, units**	37 ± 12.6, 37 [29.3, 46.9]	44.1 ± 12.7, 47.2 [36.8, 53.8]	0.002	38.5 ± 15.6, 34.9 [27.7, 46.4]	50.3 ± 26.7, 46.1 [33.1, 56.6]	0.002

Data expressed as mean ± SD, median [IQR] unless otherwise specified. *n, number of individuals; HCL, hybrid closed loop; GMI, Glucose Management Indicator; TIR, Time in range; TAR, time above range; TBR, time below range; HbA1c, glycated haemoglobin; CGM, Continuous glucose monitor; MDI, Multiple daily dose insulin; CSII, Continuous subcutaneous insulin infusion; SAP, sensor augmented pump therapy. n= 160 unless otherwise specified*

Supplementary table 5: Baseline and post-HCL outcome measures compared within ethnic groups.

Characteristic, unit of measure *(n)*	White (n=117)			Non-white (n=43)		
	Baseline	Post HCL	p-value	Baseline	Post HCL	p-value
**CGM metrics**						
**GMI, %**	7.6 ± 0.8, 7.6 [7.1, 8.1]	7.2 ± 0.6, 7.1 [6.9, 7.4]	<0.001	7.7 ± 0.7, 7.7 [7.1, 8.1]	7.3 ± 0.6, 7.2 [6.9, 7.5]	<0.001
**TAR, level 2 (>13.9 mmol/L, >250mg/dl,) %**	16.4 ± 14, 11 [6.5, 23]	8.4 ± 9.2, 6 [3, 12]	<0.001	18.8 ± 12, 17 [8, 25]	11.2 ± 9.8, 8 [5,14]	<0.001
**TAR, level 1 (10-13.9 mmol/L,180-250mg/dl) %**	27.5 ± 8.6, 28 [23, 33]	22 ± 7, 22 [17, 26.5]	<0.001	28.2 ± 5.7, 27 [25, 31]	21 ± 5.2, 21 [18, 25]	<0.001
**TIR, (3.9-10 mmol/L, 70-180mg/dl) %**	53.4 ± 17, 53 [42, 66]	68.3 ± 12.9, 70 [62, 76.5]	<0.001	50.7 ± 13.1, 51 [43, 61]	66.7 ± 12.9, 68 [60, 75]	<0.001
**TBR, level 1 (3-3.9 mmol/L, 70-54mg/dl) %**	1.9 ± 2.2, 1[0,3]	1.2 ± 1.1, 1[0,2]	<0.001	2 ± 2.3, 1 [0,3]	1.1 ± 1, 1 [0,2]	0.023
**TBR, level 2 (<3 mmol/L, 54mg/dl) %**	0.4 ± 0.9, 0 [0,0]	0.2 ± 0.6, 0 [0,0]	0.126	0.4 ± 0.9, 0 [0,1]	0.1 ± 0.4, 0 [0,0]	0.104
**Average glucose, mmol/L**	10.2 ± 1.5. 9.8 [8.8,11]	9 ± 1.3, 8.9 [8.4, 9.6]	<0.001	10.2 ± 1.5, 10.1 [8.7, 11.1]	9.2 ± 1.4, 9 [8.4, 9.8]	<0.001
**Coefficient of variation, %**	36±6.2, 35.5 [33.5, 39.6]	33 ± 4.4, 32.7 [29.9, 35.9]	<0.001	37.1 ± 6.2, 37.1 [32.7, 42.4]	35.9 ± 4.5, 35.8 [33.7, 39.2]	0.115
**Laboratory HbA1c, anthropometrics and insulin dosing**						
**HbA1c % *(n)***	7.7 ± 1.2, 7.4, [6.6, 8.3] *(n=102, 87%)*	7.2 ± 0.9, 7.1, [6.6, 7.6]	<0.001	7.9 ± 0.9, 7.9 [6.9, 8.6] *(n=37, 86%)*	7.2 ± 0.6, 7.2 [6.9, 7.6]	<0.001
**Weight, kg *(n)***	74.7 ± 13.2, 73.7 [65, 86.8] *(n=103, 87%)*	75.6 ± 14.3, 73.5 [64.5, 86.4]	0.071	75.5 ± 13.9, 70.8 [67, 84.5] *(n=37, 86%)*	77.1 ± 15.7, 74.4 [67.5, 85.9]	0.023
**BMI, kg/m^2 *(n)***	25.8 ± 4, 25.5 [23.4, 27.8] *(n=103, 87.2%)*	25.9 ± 4.2, 25.4 [23.1, 28.5]	0.497	26.8 ± 3.8, 26.7 [24.3, 28.4] *(n=37, 86%)*	27.2 ± 4.7, 26.8 [24.1, 29.2]	0.143
**Total daily insulin dose, units *(n)***	37 ± 12.6, 37 [29.3, 46.9]	38.5 ± 15.6, 34.9 [27.7, 46.4]	0.327	44.1 ± 12.7, 47.2 [36.8, 53.8]	50.3 ± 26.7, 46.1 [33.1, 56.6]	0.220

Data expressed as mean ± SD, median [IQR] unless otherwise specified. *GMI, Glucose Management Indicator; HCL, Omnipod 5 system; TIR, Time in range; TAR, time above range; TBR, time below range; HbA1c, glycated haemoglobin; CGM, Continuous glucose monitor; MDI, Multiple daily dose insulin; CSII, Continuous subcutaneous insulin infusion; SAP, sensor augmented pump therapy.*

Supplementary table 6: Baseline and post-HCL outcome measures compared between socioeconomic status (SES) groups.

**Characteristic, unit of measure *(n)***	**Baseline**			**Post-HCL**		
	Lower SES (IMD ≤ 5; n=94)	Higher SES (IMD >5; n=66)	p-value	Lower SES (IMD ≤ 5; n=94)	Higher SES (IMD >5; n=66)	p-value
**CGM metrics**						
**GMI, %**	7.7 ± 0.7, 7.6 [7.1, 8.2]	7.6 ± 0.8, 7.5 [7.1, 7.9]	0.117	7.3 ± 0.5, 7.2 [6.9, 7.5]	7.1 ± 0.6, 7 [6.8, 7.4]	0.042
**TAR, level 2 (>13.9 mmol/L, >250mg/dl,) %**	18.4 ± 13.6, 15 [7.8, 28.3]	15.1 ± 13.2, 11 [6.88, 20.3]	0.088	10 ± 8.8, 8 [4.8, 12.3]	8 ± 10.1, 5 [3,10]	0.014
**TAR, level 1 (10-13.9 mmol/L,180-250mg/dl) %**	28.1 ± 7.8, 27 [24, 33]	27.1 ± 8.2, 27.5 [23, 33.3]	0.540	21.9 ± 5.9, 22 [18, 25.3]	21.2 ± 7.4, 22 [16, 25]	0.506
**TIR, (3.9-10 mmol/L, 70-180mg/dl) %**	50.9 ± 15.5, 51 [41, 63]	55.2 ± 16.5, 5 [47, 66]	0.048	66.9 ± 11.9, 71.5 [63, 77.5]	69.4 ± 13.7, 68 [60, 75]	0.082
**TBR, level 1 (3-3.9 mmol/L, 70-54mg/dl) %**	1.9 ± 2, 1[0,3]	2 ± 2.5, 1 [0,3]	0.595	1.1 ± 1, 1[0,2]	1.2 ± 1.2, 1 [0,2]	0.869
**TBR, level 2 (<3 mmol/L, 54mg/dl) %**	0.3 ± 0.8, 0 [0,0]	0.5 ± 1.1, 0[0,0]	0.329	0.2 ± 0.5, 0 [0,0]	0.2 ± 0.5, 0[0,0]	0.639
**Average glucose, mmol/L**	10.2 ± 1.7, 10 [8.9, 11.3]	10.2 ± 3.7, 9.7 [8.7, 10.6]	0.130	9.2 ± 1.2 9.1 [8.4, 9.7]	8.9 ± 1.4, 8.6 [8, 9.4]	0.039
**Coefficient of variation, %**	36.7 ± 5.8, 35.6 [32.8, 41.1]	35.7 ± 6.7, 35.9 [33.5,39.4]	0.787	34.6 ± 4.7, 34.8 [31.5 37.5]	32.6 ± 4.2, 32.9 [29.3, 35.5]	0.012
**Laboratory HbA1c, anthropometrics and insulin dosing**						
**HbA1c % *(n, %)***	7.8 ± 1.1, 7.7 [6.8, 8.8] *(n=83, 88%)*	7.6 ± 1.2, 7.4 [6.7, 8.1] *(n=56, 85%)*	0.154	7.3 ± 0.7, 7.2, [6.7, 7.7]	7.1 ± 0.9, 7.1 [6.5, 7.6]	0.155
**Weight, kg *(n, %)***	75.5 ± 14.3, 71.9 [65.5, 86.8] *(n=83, 88%)*	74± 12.3, 72.5 [66 81.4] *(n=57, 85%)*	0.681	76.8 ± 15.5, 73.5 [66, 87]	74.9 ± 13.4, 7.5 [64.6, 82.9]	0.481
**BMI, kg/m^2 *(n, %)***	26.1 ± 4, 25.5 [23.8, 28.1] *(n=83, 88%)*	26 ± 4, 25.4 [23.7, 28.2] *(n=57, 85%)*	0.850	26.3 ± 4.4, 25.5 [23.6, 28.4]	26.1 ± 4.3, 25.8 [23.6, 28.4]	0.747
**Total daily insulin dose, units**	40.1 ± 13.2, 38 [30.9, 48]	37.3 ± 12.7, 35.5 [29, 43.2]	0.184	43.3 ± 22.8, 39.9 [28, 526]	39.3 ± 14.6, 36.6 [29.5, 46.2]	0.497

Data expressed as mean ± SD, median [IQR] unless otherwise specified. *n, number of individuals; SES, socioeconomic status; IMD, Index of multiple deprivation, HCL, hybrid closed loop; GMI, Glucose Management Indicator; TIR, Time in range; TAR, time above range; TBR, time below range; HbA1c, glycated haemoglobin; CGM, Continuous glucose monitor; MDI, Multiple daily dose insulin; CSII, Continuous subcutaneous insulin infusion; SAP, sensor augmented pump therapy. n= 160 unless otherwise specified*

Supplementary table 7: Baseline and post-HCL within socioeconomic status (SES) groups.

**Characteristic, unit of measure *(n)***	**Lower SES (IMD ≤ 5; n=94)**			**Higher SES (IMD >5; n=66)**		
	Baseline	Post HCL	p-value	Baseline	Post HCL	p-value
**CGM metrics**						
**GMI, %**	7.7 ± 0.7, 7.6 [7.1, 8.2]	7.3 ± 0.5, 7.2 [6.9, 7.5]	<0.001	7.6 ± 0.8, 7.5 [7.1, 7.9]	7.1 ± 0.6, 7 [6.8, 7.4]	<0.001
**TAR, level 2 (>13.9 mmol/L, >250mg/dl,) %**	18.4 ± 13.6, 15 [7.8, 28.3]	10 ± 8.8, 8 [4.8, 12.3]	<0.001	15.1 ± 13.2, 11 [6.88, 20.3]	8 ± 10.1, 5 [3,10]	<0.001
**TAR, level 1 (10-13.9 mmol/L,180-250mg/dl) %**	28.1 ± 7.8, 27 [24, 33]	21.9 ± 5.9, 22 [18, 25.3]	<0.001	27.1 ± 8.2, 27.5 [23, 33.3]	21.2 ± 7.4, 22 [16, 25]	<0.001
**TIR, (3.9-10 mmol/L, 70-180mg/dl) %**	50.9 ± 15.5, 51 [41, 63]	66.9 ± 11.9, 71.5 [63, 77.5]	<0.001	55.2 ± 16.5, 5 [47, 66]	69.4 ± 13.7, 68 [60, 75]	<0.001
**TBR, level 1 (3-3.9 mmol/L, 70-54mg/dl) %**	1.9 ± 2, 1[0,3]	1.1 ± 1, 1[0,2]	<0.001	2 ± 2.5, 1 [0,3]	1.2 ± 1.2, 1 [0,2]	0.01
**TBR, level 2 (<3 mmol/L, 54mg/dl) %**	0.3 ± 0.8, 0 [0,0]	0.2 ± 0.5, 0 [0,0]	0.271	0.5 ± 1.1, 0[0,0]	0.2 ± 0.5, 0[0,0]	0.052
**Average glucose, mmol/L**	10.2 ± 1.7, 10 [8.9, 11.3]	9.2 ± 1.2 9.1 [8.4, 9.7]	<0.001	10.2 ± 3.7, 9.7 [8.7, 10.6]	8.9 ± 1.4, 8.6 [8, 9.4]	<0.001
**Coefficient of variation, %**	36.7 ± 5.8, 35.6 [32.8, 41.1]	34.6 ± 4.7, 34.8 [31.5 37.5]	0.003	35.7 ± 6.7, 35.9 [33.5,39.4]	32.6 ± 4.2, 32.9 [29.3, 35.5]	<0.001
**Laboratory HbA1c, anthropometrics and insulin dosing**						
**HbA1c % *(n)***	7.7 ± 1.2, 7.4, [6.6, 8.3] *(n=102, 87%)*	7.2 ± 0.9, 7.1, [6.6, 7.6]	<0.001	7.9 ± 0.9, 7.9 [6.9, 8.6] *(n=37, 86%)*	7.2 ± 0.6, 7.2 [6.9, 7.6]	<0.001
**Weight, kg *(n)***	75.5 ± 14.3, 71.9 [65.5, 86.8] *(n=83, 88%)*	76.8 ± 15.5, 73.5 [66, 87]	0.024	74± 12.3, 72.5 [66 81.4] *(n=57, 85%)*	74.9 ± 13.5, 7.5 [64.6, 82.9]	0.152
**BMI, kg/m^2 *(n)***	26.1 ± 4, 25.5 [23.8, 28.1] *(n=83, 88%)*	26.3 ± 4.4, 25.5 [23.6, 28.4]	0.175	26 ± 4, 25.4 [23.7, 28.2] *(n=57, 85%)*	26.1 ± 4.3, 25.8 [23.6, 28.4]	0.654
**Total daily insulin dose, units *(n)***	40.1 ± 13.2, 38 [30.9, 48]	43.3 ± 22.8, 39.9 [28, 526]	0.336	37.3 ± 12.7, 35.5 [29, 43.2]	39.3 ± 14.6, 36.6 [29.5, 46.2]	0.245

Data expressed as mean ± SD, median [IQR] unless otherwise specified. *n, number of individuals; SES, socioeconomic status; IMD, Index of multiple deprivation, HCL, hybrid closed loop; GMI, Glucose Management Indicator; TIR, Time in range; TAR, time above range; TBR, time below range; HbA1c, glycated haemoglobin; CGM, Continuous glucose monitor; MDI, Multiple daily dose insulin; CSII, Continuous subcutaneous insulin infusion; SAP, sensor augmented pump therapy. n= 160 unless otherwise specified*
